# Functional similarity and molecular divergence of a novel reproductive transcriptome in two male-pregnant *Syngnathus* pipefish species

**DOI:** 10.1002/ece3.763

**Published:** 2013-09-20

**Authors:** Clayton M Small, April D Harlin-Cognato, Adam G Jones

**Affiliations:** 1 Department of Biology, Texas A&M UniversityCollege Station, Texas, 77843, USA; 2 Institute of Ecology and Evolution, University of OregonEugene, Oregon, 97403, USA; 3 Department of Zoology, Michigan State UniversityEast Lansing, Michigan, 48824, USA

**Keywords:** Male pregnancy, molecular evolution, pyrosequencing, RNA-seq, Syngnathidae, transcriptome

## Abstract

Evolutionary studies have revealed that reproductive proteins in animals and plants often evolve more rapidly than the genome-wide average. The causes of this pattern, which may include relaxed purifying selection, sexual selection, sexual conflict, pathogen resistance, reinforcement, or gene duplication, remain elusive. Investigative expansions to additional taxa and reproductive tissues have the potential to shed new light on this unresolved problem. Here, we embark on such an expansion, in a comparison of the brood-pouch transcriptome between two male-pregnant species of the pipefish genus *Syngnathus*. Male brooding tissues in syngnathid fishes represent a novel, nonurogenital reproductive trait, heretofore mostly uncharacterized from a molecular perspective. We leveraged next-generation sequencing (Roche 454 pyrosequencing) to compare transcript abundance in the male brooding tissues of pregnant with nonpregnant samples from Gulf (*S. scovelli*) and dusky (*S. floridae*) pipefish. A core set of protein-coding genes, including multiple members of astacin metalloprotease and c-type lectin gene families, is consistent between species in both the direction and magnitude of expression bias. As predicted, coding DNA sequence analysis of these putative “male pregnancy proteins” suggests rapid evolution relative to nondifferentially expressed genes and reflects signatures of adaptation similar in magnitude to those reported from *Drosophila* male accessory gland proteins. Although the precise drivers of male pregnancy protein divergence remain unknown, we argue that the male pregnancy transcriptome in syngnathid fishes, a clade diverse with respect to brooding morphology and mating system, represents a unique and promising object of study for understanding the perplexing evolutionary nature of reproductive molecules.

## Introduction

An unprecedented appreciation of the complexity of interactions between the sexes during courtship and mating has spurred keen interest in the evolution of molecules involved in reproduction (Swanson and Vacquier 2002a,b; Clark et al. 2006). On one level, the reproductive enterprise appears to be a relatively straightforward cooperative process, as both sexes often make nontrivial contributions to the common goal of producing offspring (Clutton-Brock 1991; Gross 2005). On closer inspection, however, we see that reproduction involves numerous cooperative and antagonistic interactions at various levels of biological organization, ranging from behavior at the level of whole organisms to processes involving molecules associated with gametes. In terms of these latter molecular interactions, a number of different factors could contribute to adaptive evolution of genes encoding products associated with reproduction. For example, proteins expressed by eggs and sperm can be involved in species recognition and consequently play a central role in the evolution of reproductive isolation in some lineages (Lee and Vacquier 1992; Palumbi 1994; Metz and Palumbi 1996; Swanson and Vacquier 2002a). Proteins associated with gametes can also be targets of sexual selection within species, a process that could in principle drive very rapid evolution of reproductive genes (Wyckoff et al. 2000; Birkhead and Pizzari 2002; Torgerson et al. 2002). Of course, the reproductive interests of the sexes seldom coincide perfectly, and this situation results in sexual conflict (Trivers 1972; Parker 1979; Chapman et al. 2003; Arnqvist and Rowe 2005), another mechanism that can produce rapid evolution of reproductive proteins as males and females are drawn into an evolutionary arms race (Chapman et al. 1995; Swanson et al. 2004; Fricke et al. 2009). Other possible factors involved in reproductive protein evolution, such as relaxed constraints, gene duplication, or protection against pathogens, are discussed by Swanson and Vacquier (2002b).

One notable pattern in the study of reproductive proteins thus far is that most studies have focused on the molecular evolution of proteins present in sperm or transferred along with sperm during mating (Swanson and Vacquier 2002a,b; Turner and Hoekstra 2008). This bias is understandable, because sexual selection acts more strongly on males than on females in most types of organisms. Studies of sperm-associated proteins have revealed rapid evolution and evidence for positive selection, as demonstrated by a high ratio of nonsynonymous to synonymous nucleotide substitutions (i.e., *d*_N_/*d*_S_) in many of the loci associated with male reproduction. Against this backdrop, a growing body of literature has begun to characterize patterns of molecular evolution in ova and other reproductive tissues. For instance, egg-specific proteins in abalone show a strong signature of positive selection, which can be interpreted as evidence for coevolution of proteins present in eggs with the rapidly evolving sperm proteins (Galindo et al. 2003; Aagaard et al. 2013). Other studies of egg-specific reproductive proteins have found similar evidence for elevated rates of positive selection (Swanson et al. 2001a; Turner and Hoekstra 2006; Claw and Swanson 2012). More recently, a few studies have branched out to examine patterns of molecular evolution in genes expressed in other reproductive structures, such as the placenta in mammals and fishes of the genus *Poeciliopsis* and the stamen of plants, and have also found evidence for elevated signatures of positive selection (O'Neill et al. 2007; Chuong et al. 2010; Yang and Wang 2013). Thus, the balance of evidence suggests that proteins associated with reproduction tend to have higher rates of molecular evolution, with stronger signatures of positive selection, compared with loci chosen at random from the genome. These patterns of molecular evolution are intriguing, but the field would benefit from additional studies focusing on the reproductive genes from a wider array of taxa and reproductive structures to confirm the generality of these observations.

One taxonomic group with the potential to make a contribution to the study of reproductive protein evolution is the fish Family Syngnathidae (pipefishes, seahorses, and seadragons), a group that has evolved the unusual habit of male pregnancy (Dawson 1985). From a reproductive tissue standpoint, the syngnathid fishes are especially exciting, because some taxa in this group have evolved a complex, specialized brood pouch that develops on the ventral surface of the male and into which females deposit eggs during mating (Herald 1959; Wilson et al. 2001). While all syngnathid fishes carry the developing embryos on the males, not all of them have a brood pouch. Rather, some species of pipefishes and seadragons carry the eggs attached to the ventral surface of the male with no outer covering. In addition, the brood-pouch structure is thought to have evolved at least twice in the Syngnathidae (Wilson et al. 2003; Wilson and Rouse 2010; Wilson and Orr 2011), so it provides an opportunity to study convergent evolution in a phylogenetic context. Thus, the brood pouch of the Family Syngnathidae provides a novel reproductive tissue, which has an interesting evolutionary history and has the potential to contribute to a better understanding of the role of positive selection in the evolution of reproductive proteins.

**Figure 1:**
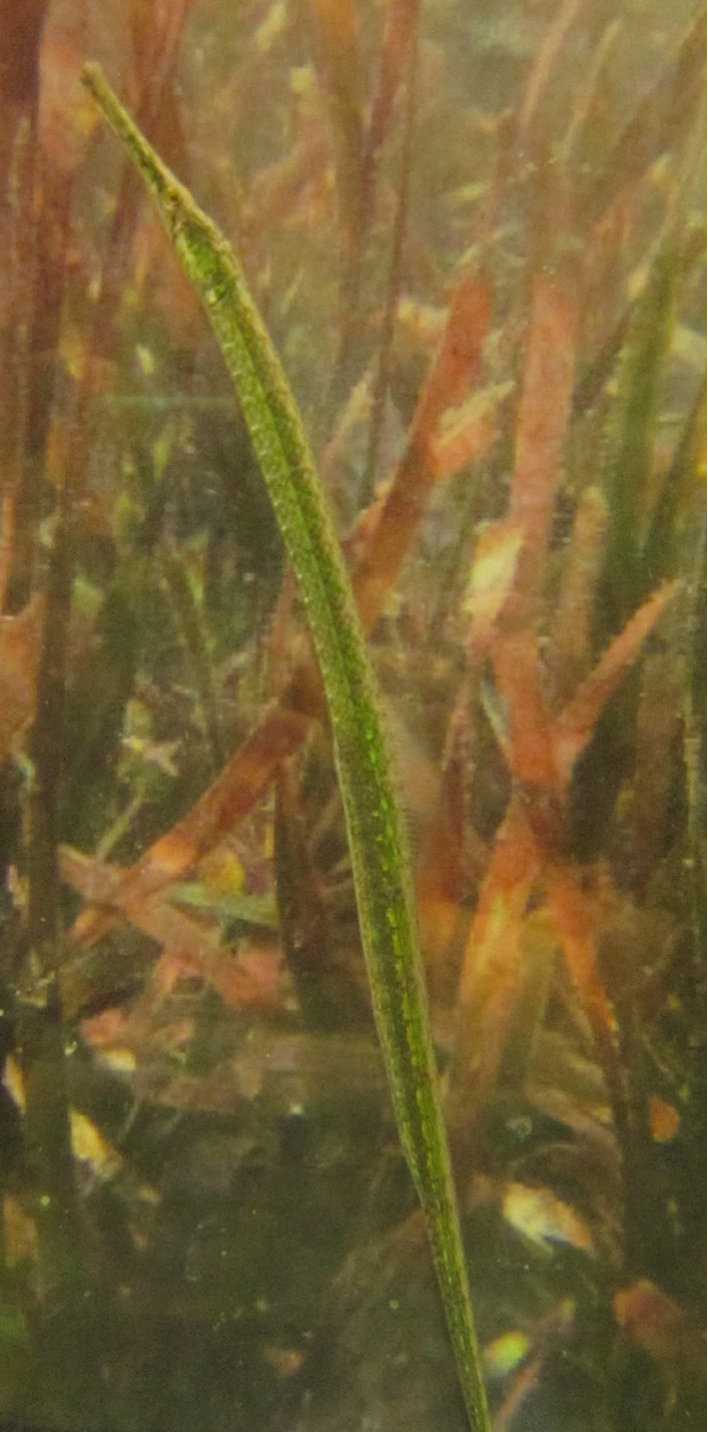
An adult, nonpregnant male *S. floridae*. The animal's brood pouch is located posterior to the urogenital opening (near the center of the dorsal fin), and extends down the tail. Photo courtesy of Sarah Flanagan.

In this study, we focus on the evolution of brood-pouch proteins expressed during male pregnancy in two species of pipefish, *Syngnathus scovelli* and *S. floridae* (Fig. 1). Male pipefish of the genus *Syngnathus* possess a brood pouch with a ventral seam (Herald 1959), which remains completely closed during the pregnancy. Both of these species are sex-role reversed in the sense that sexual selection acts more strongly on females than on males (Jones and Avise 1997a,b; Jones et al. 2001; Mobley and Jones 2007, 2009). Males have complete confidence of paternity, because the female transfers unfertilized eggs during mating and fertilization occurs within the pouch (Jones and Avise 1997b). Males of *S. scovelli* typically mate with one female per pregnancy (Jones and Avise 1997a), whereas males of *S. floridae* typically mate with multiple females per pregnancy (Jones and Avise 1997b; Mobley and Jones 2007, 2009). During the pregnancy, males transfer nutrients to the offspring through highly vascularized paternal tissue, which proliferates and surrounds each embryo within the brood pouch (Ripley and Foran 2006, 2009; Kvarnemo et al. 2011). In *S. scovelli*, the number of offspring born is often substantially less than the number of eggs initially received (Paczolt and Jones 2010), and work involving the congener *S. typhle* suggests that males can absorb nutrients from their broods (Sagebakken et al. 2010). Thus, the brood pouch in *Syngnathus* could be a mediator of differential allocation, postcopulatory sexual selection, or sexual conflict (Paczolt and Jones 2010). These considerations lead to the prediction that the brood pouch will be involved in the coevolutionary arms race that occurs between males and females, and we consequently expect an elevated rate of molecular evolution for genes involved in male pregnancy compared with other genes in the genome.

In this study, we have two major goals. The first is to characterize the “male pregnancy transcriptome” for the first time in the Family Syngnathidae using next-generation approaches to sequence mRNA from genes expressed in the brood pouch. Two previous studies have sequenced a handful of expressed sequence tags from male seahorses (*Hippocampus kuda* and *H. comes*), but neither of these studies, which predated the widespread use of next-generation sequencing, were able to compare levels of expression between pregnant and nonpregnant males (Zhang et al. 2003; Melamed et al. 2005). Our second goal is to compare male pregnancy transcriptomes between *S. scovelli* and *S. floridae*, two recently diverged pipefish. This comparison allows us to explore the hypothesis that genes involved in male pregnancy (i.e., those that are up- or down-regulated in the brood pouch during pregnancy) experience a stronger signature of positive selection on average compared with genes that are not differentially expressed during the pregnancy. The results of this study shed light on the evolution of the transcriptome of the brood pouch, a novel type of male reproductive tissue, and pave the way for future, broader comparisons of the evolution of male pregnancy in the Family Syngnathidae.

## Material and Methods

### Sample preparation and Roche 454 GS FLX sequencing

We obtained pregnant, adult male Gulf pipefish (*S. scovelli*) and dusky pipefish (*S. floridae*) in July 2008 from seagrass beds in the Gulf of Mexico (Redfish Bay, TX). Animals were housed in 100-L volumes of seawater in biologically filtered tanks at 25°C for 48 h following capture, to allow males late in pregnancy to give birth. Consequently, all nonpregnant males used for the study had given birth in the preceding 48 h. We ensured that all pregnant males used for the study were in early stages of pregnancy (the first trimester). That is, they were brooding embryos at or before “state 2” of Ripley and Foran's pipefish developmental series (Ripley and Foran 2006). We realize that differences in gene expression among males may be partially due to environmental variation in the field. Although a completely controlled experiment in which all individuals are raised in the laboratory could in principle minimize this variation, a resulting tradeoff could be the potential for artificial, laboratory-induced gene expression patterns. Each male was euthanized immediately prior to dissection with a lethal dose of MS222 buffered to physiological pH. All brooding structures, including the pouch and ventrally suspended epithelial tissues, were carefully and quickly excised from each animal, rinsed with sterile water, and snap frozen at −80°C. Embryos were cautiously removed and discarded from pregnant males before tissue preparations.

We homogenized the frozen brooding tissues by pestle and isolated total RNA from each sample using TRIzol® Reagent (Life Technologies, Carlsbad, CA), in accordance with the manufacturer's standard protocol. At this stage, 11 μg total RNA from five pregnant and five nonpregnant *S. scovelli* and *S. floridae* was combined within species and pregnancy status to create four pools, as was necessary to obtain sufficient material (55 μg per pool) for mRNA selection with the Oligotex® mRNA Mini Kit (Qiagen, Venlo, Netherlands). We used 720 ng of the resulting mRNA from each pool as template for cDNA synthesis with the SMART™ cDNA Library Construction Kit (Clontech, Mountain View, CA). In general, the manufacturer's reagents and long distance (LD) PCR guidelines were used, but a modified CDSIII/3' cDNA Synthesis Primer (5'- TAG AGG CCG AGG CGG CCG ACA TGT TTT GTT TTT TTT TCT TTT TTT TTT VN -3') and SuperScript® II Reverse Transcriptase (Life Technologies) were used in place of kit reagents. After a PCR cycle number titration, we optimized our protocol at 17 cycles of PCR, and all steps after LD PCR were completed as described in the SMART™ protocol, without cloning. We sent 15 μg of cDNA from each of the four libraries (pregnant *S. scovelli*, nonpregnant *S. scovelli*, pregnant *S. floridae*, nonpregnant *S. floridae*) to the Michigan State University Research Technology Support Facility, where the libraries were multiplexed and sequenced in two runs on a 454 GS FLX® sequencer using Titanium® chemistry (Roche, Basel, Switzerland).

### De novo transcriptome assembly, differential expression analysis, and GO term enrichment analysis

The 454 reads were trimmed to remove low-quality regions, polyA tails, and cDNA synthesis artifacts using the highly customizable pipeline *clean_reads*, from the *ngs_backbone* suite of bioinformatics tools (Blanca et al. 2011). After discarding all cleaned reads < 50 nt, we performed two de novo transcriptome assemblies (one for each species) using the CLC Genomics Workbench® version 4.8 (CLC bio, Aarhus, Denmark). After varying assembly parameter values to assess optimality, we set the k-mer size at 20, selected the remapping option with similarity criterion set at 0.97, specified a minimum contig size of 200 nt, and used a majority rule consensus to decide the state of bases that were variable among reads (either due to polymorphism or sequencing error). We also included in the final assembly only those contigs to which at least one read was uniquely mappable.

Orthologous transcripts were identified using a “reciprocal best BLAST hit” criterion (Rivera et al. 1998), wherein the BLAST hit (Altschul et al. 1990) for each search with the highest bit-score is used to establish the “best hit.” This approach stipulates that orthologous pairs are only obtained in the event that top hits from BLAST searches in both directions are reciprocally consistent. This approach is comparable in reliability to more phylogenetically intensive methods (Altenhoff & Dessimov 2009) and is an alternative to more rigorous criteria which require genomic references from focal or closely related species.

We estimated library-specific, per-contig transcript abundance by mapping sequencing reads from a given library back to its respective species' assembly with the RNA-seq module in the CLC Genomics Workbench®. The number of uniquely mapped reads per contig was then used as a proxy for transcript abundance, and we used the R package DEGseq (Wang et al. 2010) to statistically compare transcript abundance between pregnant and nonpregnant libraries separately for the two species. In particular, we used the MA plot with random sampling approach (MARS) in DEGseq to assess, for each contig, whether there was evidence for a difference between libraries in the proportion of reads mapped uniquely. This approach takes into account differences in library size when inferring differential expression. We used the p-values from a subset of the contigs to categorize transcripts for each species as “pregnancy-enriched,” “pregnancy-depressed,” or “non-differentially expressed.” This subset included only the contigs for which an ortholog in the other species was identified, because we were interested in comparing sets of genes represented in both species. Furthermore, because we wanted to limit the impact of low sequencing coverage on differential expression inferences, the subset only included contigs to which at least 10 reads (from either the pregnant or nonpregnant library) mapped uniquely. These filtering criteria resulted in a subset of 2051 contigs for *S. scovelli* and 1090 contigs for *S. floridae*. After applying a false discovery rate of 0.05 (Benjamini and Hochberg 1995) to the DEGseq *P*-values for these groups of contigs, we considered a transcript “pregnancy-enriched” if it demonstrated a normalized pregnant-to-nonpregnant fold change >1.5, and “pregnancy-depressed” if it demonstrated a normalized nonpregnant-to-pregnant fold change >1.5. Alternatively, we considered a transcript “evenly expressed” if it did not meet the false discovery rate criterion for differential expression and it demonstrated a normalized pregnant-to-nonpregnant fold change between 1.3^−1^ and 1.3.

We characterized the functional composition of the three above expression categories for both species using the software Blast2GO (Gotz et al. 2008) for gene ontology (GO) term analysis. Briefly, we used top BLASTx hits to extract and count the number of “level 2 molecular function” GO terms for contigs in each expression category. We considered “molecular function,” as opposed to “biological process” terms to be the most informative for annotation because it is unclear how biological process terms such as “response to stimulus” and “reproduction,” for example, should be interpreted in the context of male brooding biology, a phenomenon removed from the organismal processes on which the original GO annotations are based. That is, a more reductionist set of annotations including molecular function terms such as “structural activity” and “catalytic activity” are general but necessarily more relevant to the molecular and physiological processes that facilitate male pregnancy. For each species, we tested for differences among expression groups in the frequency of 15 terms, using 3 × 2 *G*-tests of independence with a Williams's correction (Sokal and Rohlf 1994) and 0.05 false discovery rate adjustment (Benjamini and Hochberg 1995).

### Alignment of orthologous protein-coding sequences and molecular evolutionary analyses

To compare rates of molecular evolution among pregnancy-enriched, pregnancy-depressed, and nondifferentially expressed protein-coding sequences, we first restricted our list of orthologs to those that fell into the same one of the three above expression groups for both species. We randomly selected 50 of the 142 orthologous pairs from the “evenly expressed” set as a constitutively expressed “control” panel. These groupings likely represent biologically meaningful examples of the three expression categories, given intersecting evidence from the two pipefish species. We aligned each orthologous pair of contigs from this limited set of expression groups with the help of a reference protein-coding sequence obtained from BLASTx (Camacho et al. 2009) queries of the NCBI nonredundant protein sequence database, using the alignment software ClustalW (Thompson et al. 1994). We manually inspected all sequence alignments, trimming untranslated regions and any unaligned overhanging sequence. We also excluded from further analysis any coding sequence alignment shorter than 210 nt (70 amino acids) to avoid biases associated with the maximum likelihood estimation of substitution rates for especially short sequences.

Pairwise *S. floridae*-*S. scovelli* estimates of *d*_N_, *d*_S_, and *d*_N_/*d*_S_ were estimated via maximum likelihood from 19 pregnancy-enriched, 69 pregnancy-depressed, and the random sample of 50 nondifferentially expressed coding sequence alignments using the program codeml (runmode -2) within PAML 4.5 (Yang 1997). The *d*_N_/*d*_S_ ratio is interpreted as a measure of amino acid divergence standardized by the nearly neutral “background” rate of silent substitution (Yang and Bielawski 2000). We compared *d*_N_, *d*_S_, and *d*_N_/*d*_S_ among the three expression groups using Kruskal–Wallis tests and performed post hoc pairwise comparisons using Mann–Whitney U-tests. Because it is possible to obtain maximum likelihood estimates of *d*_N_/*d*_S_ that are undefined when *d*_S_ is equal to zero (making interpretation difficult), we excluded from analysis all *d*_N_/*d*_S_ estimates for genes lacking synonymous substitutions. All statistical tests for this study were either performed by hand or using JMP Pro® version 9 (SAS Institute Inc., Cary, NC), unless mentioned otherwise.

## Results

### 454 sequencing and de novo transcriptome assembly

We obtained 206863, 373152, 182750, and 153971 high-quality reads from the *S. scovelli* pregnant, *S. scovelli* nonpregnant, *S. floridae* pregnant, and *S. floridae* nonpregnant libraries, respectively. Mean read length across all four libraries was 277 nt. The *S. scovelli* transcriptome assembly consisted of 15424 contigs with a mean contig length of 613 nt, a median length of 498 nt, a range of 200–6272 nt, and a N50 of 661 nt. The *S. floridae* assembly consisted of 8787 contigs with a mean contig length of 581, a median length of 489 nt, a range of 200–5717 nt, and a N50 of 617 nt. These statistics are on par with *de novo* transcriptome assemblies from similar studies of teleosts in which 454 sequencing was applied (Elmer et al. 2010; Renaut et al. 2010). All contig names along with their lengths, numbers of uniquely mapped reads, normalized fold changes, and DEGseq *P*-values are included in Tables S1 and S2, corresponding to *S. scovelli* and *S. floridae*, respectively.

### Differential expression with respect to pregnancy status among orthologous transcripts

**Figure 2:**
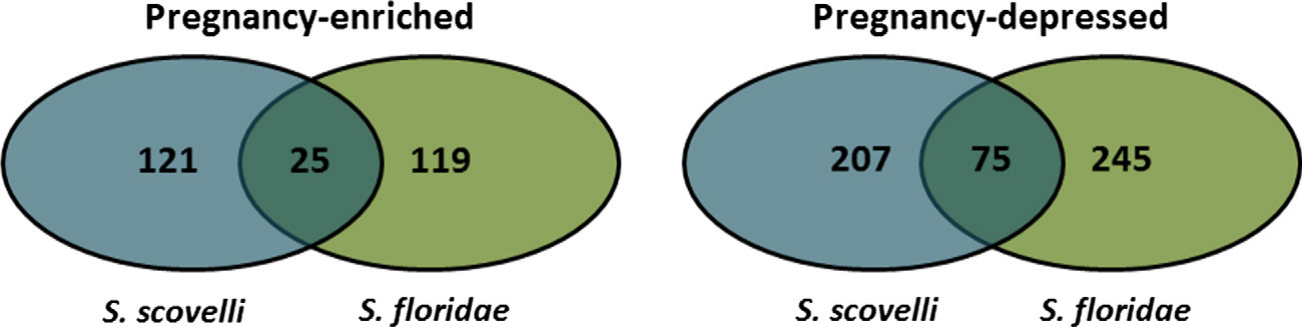
Between-species overlap of genes differentially expressed as a consequence of male pregnancy status. Venn diagrams depict the proportion of pregnancy-enriched (left) and pregnancy-depressed (right) transcripts consistent between *S. floridae* and *S. scovelli*.

**Figure 3:**
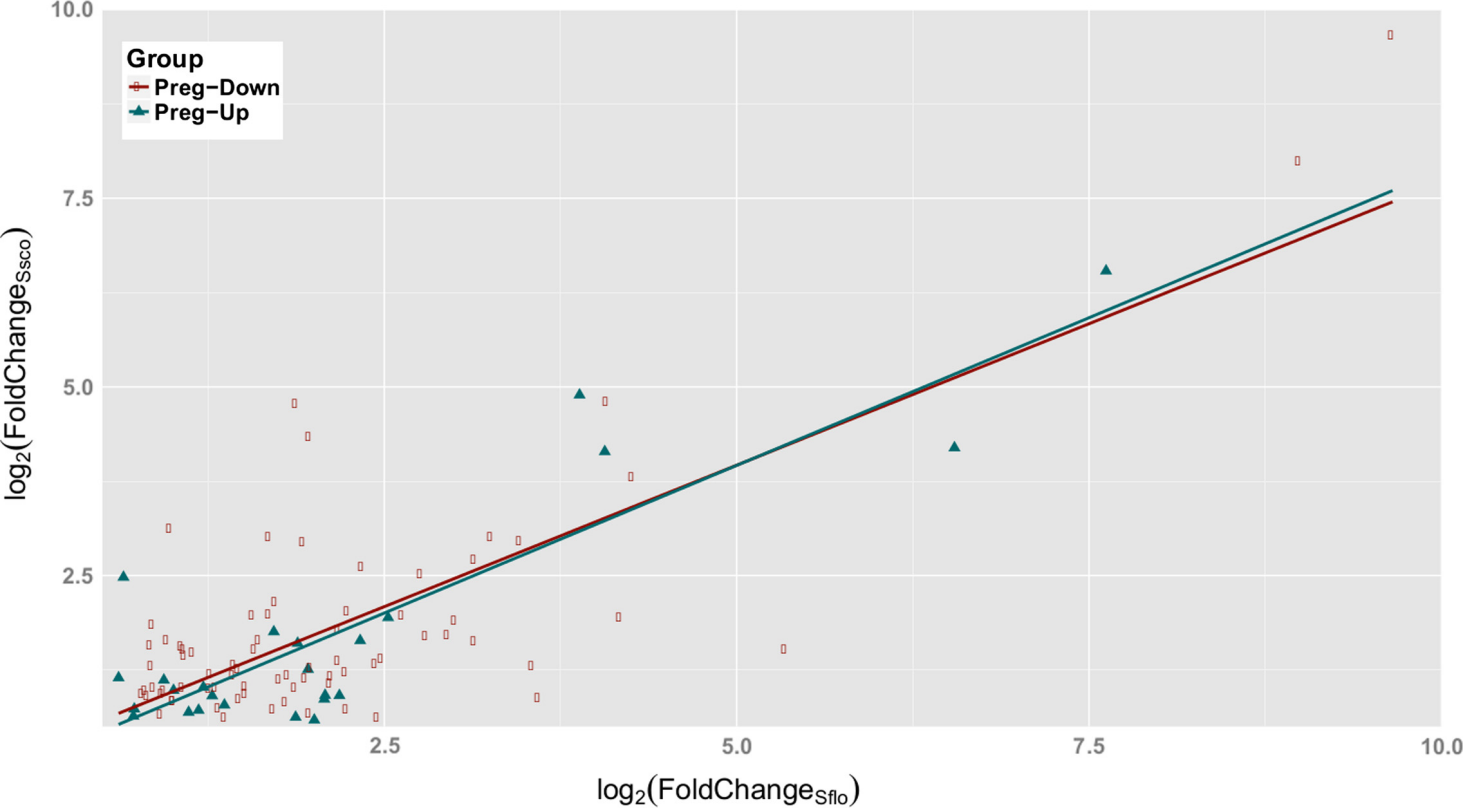
The magnitude of differential expression with respect to male pregnancy status covaries between species for both pregnancy-enriched and pregnancy-depressed transcripts. Log-transformed fold changes (normalized by total library read number) for *S. scovelli* orthologs are plotted against those for *S. floridae* orthologs. The red rectangles and least-squares regression line correspond to pregnancy-depressed transcripts, whereas the blue triangles and least-squares regression line correspond to pregnancy-enriched transcripts.

Although initial analysis of complete sets of contigs revealed hundreds of putatively differentially expressed transcripts (see Figs. S1 and S2 for DEG-seq MA-plots of *S. scovelli* and *S. floridae* data, respectively), we decided to restrict our differential expression inferences to orthologs with at least 10 uniquely mapped reads from any one library. Our reciprocal best BLAST hit screen for orthologs yielded 5508 contig pairs. Of these, 2051 *S. scovelli* contigs and 1089 *S. floridae* contigs passed this read coverage minimum. For *S. scovelli*, DEG-seq statistical tests with the false discovery rate controlled at 0.05 revealed 121 pregnancy-enriched, 207 pregnancy-depressed, and 778 evenly expressed contigs. Likewise, for *S. floridae,* we identified 119 pregnancy-enriched, 245 pregnancy-depressed, and 296 evenly expressed contigs. Between-species comparisons showed that 25 orthologous transcripts were pregnancy-enriched in both species and 75 orthologous transcripts were pregnancy-depressed in both species (Fig. 2). Tables 1 and 2 provide annotation information and normalized fold changes for each pregnancy-enriched and pregnancy-depressed transcript, respectively. Additional information for these contigs and the 50 “evenly expressed” contigs, including sequence length, number of mapped reads, and original DEGseq *P*-values, may be found in Table S3. Mean contig length (the two-species average) is not different among the three expression groups (Kruskal–Wallis test: *N*_*preg-up*_ = 25, *N*_*preg-down*_ = 75, *N*_*preg-even*_ = 50; *χ*^*2*^ = 0.5749; *P* = 0.750). The total number of uniquely mapped reads from all four libraries, however, does differ among categories (Kruskal–Wallis test: *N*_*preg-up*_ = 25, *N*_*preg-down*_ = 75, *N*_*preg-even*_ = 50; *χ*^*2*^ = 9.467; *P* = 0.009), due to a higher number of total mapped reads for pregnancy-enriched, relative to evenly expressed, transcripts (post hoc Mann–Whitney *U*-test: *Z* = 3.096; *P* = 0.002). The magnitude of differential expression (normalized fold change) is strongly concordant between species for pregnancy-enriched (*r*^*2*^ = 0.928, *P* < 0.001) and pregnancy-depressed (*r*^*2*^ = 0.863, *P* < 0.001) transcripts (Fig. 3).

**Table 1:** Blastx annotation and fold change information for the 25 orthologous transcripts overexpressed in pregnant (P.) relative to nonpregnant (N.P.) samples in both species. The sequence description, species name (in brackets), NCBI accession number, and e-value of each top blastx hit are indicated, as well as the pregnant-to-nonpregnant fold change values (normalized for library read totals) for both species

Top Blastx Hit Description	Accession #	E-value	*S.flo*. Fold (P./N.P.)	*S.sco*. Fold (P./N.P.)
Estrogen-regulated protein [*Sparus aurata*]	ACX94453.1	6.00E-40	>196.83	>92.84
PREDICTED: probable chitinase 3-like [*Callithrix jacchus*]	XP_002751292.1	2.00E-19	93.39	18.28
Patristacin [*Syngnathus scovelli*]	ABK80840.1	8.00E-122	16.73	17.66
Bactericidal permeability-increasing protein [*Paralichthys olivaceus*]	ACV74252.1	0	14.78	29.71
PREDICTED: neoverrucotoxin subunit alpha-like [*Oreochromis niloticus*]	XP_003449483.1	1.00E-172	5.76	3.85
PREDICTED: glutamine synthetase [*Oreochromis niloticus*]	XP_003438182.1	0	5.02	3.11
No Hit	NA	NA	4.54	1.88
No Hit	NA	NA	4.23	1.88
No Hit	NA	NA	4.21	1.82
Keratin, type I cytoskeletal 18 [*Dicentrarchus labrax*]	CBN80920.1	0	4.01	1.50
Annexin A5 [*Anoplopoma fimbria*]	ACQ58091.1	0	3.89	2.39
Integrin beta-4 [*Dicentrarchus labrax*]	CBN81160.1	2.00E-100	3.69	3.04
Lectin protein type II [*Hippocampus comes*]	AAQ56013.1	2.00E-46	3.66	1.54
PREDICTED: CD59 glycoprotein-like [*Oryzias latipes*]	XP_002660983.1	3.00E-05	3.29	3.38
Annexin max3 [*Oryzias latipes*]	NP_001098295.1	0	2.58	1.72
S100-like calcium- binding protein [*Sparus aurata*]	ACX94454.1	7.00E-25	2.43	1.88
PREDICTED: c-C motif chemokine 25-like [*Oreochromis niloticus*]	XP_003448528.1	8.00E-29	2.32	2.03
Hemoglobin alpha chain [*Hippocampus comes*]	AAR11385.1	6.00E-79	2.27	1.64
Ictacalcin [*Oncorhynchus mykiss*]	NP_001158643.1	2.00E-26	2.16	1.61
PREDICTED: transgelin-like isoform 2 [*Oreochromis niloticus*]	XP_003449375.1	1.00E-128	2.01	1.97
PREDICTED: clusterin-like [*Oreochromis niloticus*]	XP_003446372.1	2.00E-154	1.91	2.17
PREDICTED: decorin [*Oreochromis niloticus*]	XP_003444991.1	0	1.65	1.66
Adult beta-type globin [*Oryzias latipes*]	BAC06483.1	4.00E-82	1.65	1.56
Lectin protein type II [*Hippocampus comes*]	AAQ56013.1	2.00E-20	1.57	5.57
PREDICTED: follistatin-related protein 1-like [*Oreochromis niloticus*]	XP_003452992.1	0	1.53	2.21

**Table 2:** Blastx annotation and fold change information for the 75 orthologous transcripts overexpressed in nonpregnant (N.P.) relative to pregnant (P.) samples in both species. The sequence description, species name (in brackets), NCBI accession number, and e-value of each top blastx hit are indicated, as well as nonpregnant-to-pregnant fold change values (normalized for library read totals) for both species

Top Blastx Hit Description	Accession #	E-value	*S.flo*. Fold (N.P./P.)	*S.sco*. Fold (N.P./P.)
Patristacin [*Syngnathus scovelli*]	ABK80840.1	4.00E-86	>805.29	>812.11
Lectin protein type II [*Hippocampus comes*]	AAQ56013.1	2.00E-46	510.08	>254.19
Glucose-6-phosphate 1-dehydrogenase [*Salmo salar*]	NP_001135196.1	0	40.60	2.88
PREDICTED: transmembrane emp24 domain-containing protein 5-like [*Takifugu rubripes*]	XP_003973910.1	6.00E-42	19.17	>14.00
Unnamed protein product [*Tetraodon nigroviridis*]	CAF93928.1	7.00E-23	18.05	3.85
Tripartite motif-containing protein 39 [*Cricetulus griseus*]	EGW01417.1	7.00E-28	16.92	28.00
Myeloperoxidase [*Siniperca chuatsi*]	ABC72122.1	0	12.07	1.84
PREDICTED: 5-aminolevulinate synthase, nonspecific, mitochondrial-like [*Oreochromis niloticus*]	XP_003454600.1	0	11.73	2.48
PREDICTED: signal-transducing adaptor protein 2-like [*Oreochromis niloticus*]	XP_003439745.1	2.00E-60	11.05	7.81
Protein kinase C, delta [*Dicentrarchus labrax*]	CBN81068.1	0	9.59	8.08
PREDICTED: hematopoietic SH2 domain-containing protein homolog [*Takifugu rubripes*]	XP_003974199.1	2.00E-41	8.83	6.56
PREDICTED: NADPH oxidase organizer 1-like [*Oreochromis niloticus*]	XP_003452925.1	3.00E-112	8.83	3.10
PREDICTED: transmembrane protease serine 13-like [*Oreochromis niloticus*]	XP_003450894.1	5.00E-104	8.04	3.77
Lectin protein type I [*Hippocampus comes*]	AAQ56012.1	5.00E-35	7.73	3.29
PREDICTED: hypothetical protein LOC100712072 [*Oreochromis niloticus*]	XP_003460123.1	7.00E-13	6.94	3.24
PREDICTED: vacuolar protein sorting-associated protein 4B-like [*Oreochromis niloticus*]	XP_003437949.1	0	6.77	5.74
PREDICTED: actin-related protein 2-B-like isoform 2 [*Oreochromis niloticus*]	XP_003438377.1	0	6.20	3.95
No Hit			5.60	2.63
PREDICTED: interleukin-10 receptor subunit beta-like [*Oreochromis niloticus*]	XP_003443306.1	9.00E-49	5.48	1.54
Natural killer cell enhancing factor [*Oplegnathus fasciatus*]	BAK38717.1	5.00E-134	5.43	2.53
PREDICTED: zymogen granule membrane protein 16-like [*Oryzias latipes*]	XP_004073801.1	5.00E-23	5.08	6.14
No Hit			4.74	4.09
PREDICTED: ATP synthase subunit b, mitochondrial-like [*Oreochromis niloticus*]	XP_003444862.1	7.00E-141	4.72	1.67
PREDICTED: hypothetical protein LOC100712072 [*Oreochromis niloticus*]	XP_003460123.1	8.00E-07	4.67	2.34
PREDICTED: aldo-keto reductase family 1 member B10-like [*Oreochromis niloticus*]	XP_003450753.1	2.00E-180	4.51	3.43
PREDICTED: aldehyde dehydrogenase, mitochondrial [*Oreochromis niloticus*]	XP_003446212.1	0	4.51	2.58
PREDICTED: pituitary tumor-transforming gene 1 protein-interacting protein-like [*Oreochromis niloticus*]	XP_003442975.1	5.00E-80	4.35	2.24
Thiosulfate sulfurtransferase KAT [*Osmerus mordax*]	ACO09872.1	3.00E-50	4.33	2.11
Proteasome activator complex subunit 1 [*Oplegnathus fasciatus*]	AFO64917.1	3.00E-114	3.95	2.42
Zymogen granule membrane protein 16 precursor [*Anoplopoma fimbria*]	ACQ58296.1	1.00E-30	3.92	20.34
Selenophosphate synthetase 2 [*Danio rerio*]	NP_001004295.2	0	3.91	1.60
PREDICTED: cornifelin-like [*Oreochromis niloticus*]	XP_003453505.1	4.00E-37	3.83	2.21
Omega class glutathione S-transferase [*Oplegnathus fasciatus*]	ADY80021.1	6.00E-134	3.81	7.72
PREDICTED: sodium/potassium-transporting ATPase subunit gamma-like [*Oreochromis niloticus*]	XP_003450860.1	1.00E-15	3.67	27.47
Hypothetical protein VOLCADRAFT_30543 [*Volvox carteri f. nagariensis*]	XP_002959253.1	5.00E-05	3.65	2.03
S100-like protein [*Epinephelus bruneus*]	AEH76608.1	1.00E-05	3.52	2.26
PREDICTED: lysozyme C, milk isozyme-like [*Oryzias latipes*]	XP_004065665.1	2.00E-07	3.49	1.77
PREDICTED: HIG1 domain family member 2A-like [*Oreochromis niloticus*]	XP_003445914.1	1.00E-48	3.38	2.19
PREDICTED: LOW-QUALITY PROTEIN: redox-regulatory protein FAM213A-like [*Oryzias latipes*]	XP_004080653.1	8.00E-77	3.31	4.46
No Hit			3.29	1.66
PREDICTED: tumor necrosis factor alpha-induced protein 8-like protein 3-like [*Oreochromis niloticus*]	XP_003458234.1	8.00E-114	3.22	8.08
Charged multivesicular body protein 4c [*Dicentrarchus labrax*]	CBN82057.1	3.00E-77	3.22	3.99
Lectin protein type III [*Hippocampus comes*]	AAQ56014.1	3.00E-52	3.06	3.14
PREDICTED: serine protease 27-like [*Oreochromis niloticus*]	XP_003442612.1	4.00E-111	2.99	2.89
No Hit			2.96	3.95
PREDICTED: transaldolase-like [*Oreochromis niloticus*]	XP_003450837.1	0	2.87	2.04
PREDICTED: proteasome subunit beta-type-7-like [*Oreochromis niloticus*]	XP_003439536.1	4.00E-177	2.86	1.91
Proteasome subunit alpha type-6-like [*Oryzias latipes*]	XP_004083706.1	7.00E-165	2.77	1.83
Phosphatidylinositol transfer protein, alpha [*Mustela putorius furo*]	AES04090.1	1.00E-133	2.77	2.40
PREDICTED: 15-hydroxyprostaglandin dehydrogenase [NAD+]-like [*Oreochromis niloticus*]	XP_003456541.1	3.00E-166	2.70	2.49
Ependymin-1 precursor [*Anoplopoma fimbria*]	ACQ58356.1	3.00E-80	2.69	2.26
Selenoprotein W2 [*Artemia franciscana*]	ABS19961.1	6.00E-06	2.58	1.54
Beta-2 microglobulin isoform b2 m-2 [*Seriola quinqueradiata*]	BAI63127.1	6.00E-41	2.51	1.68
PREDICTED: protein tyrosine phosphatase type IVA 3-like [*Takifugu rubripes*]	XP_003966081.1	5.00E-99	2.46	2.03
PREDICTED: charged multivesicular body protein 5-like [*Oreochromis niloticus*]	XP_003439285.1	1.00E-134	2.41	2.29
PREDICTED: transketolase [*Oreochromis niloticus*]	XP_003448256.1	0	2.39	2.01
Alpha-class glutathione S-transferase [*Epinephelus coioides*]	ACI01805.1	1.00E-122	2.21	2.80
PREDICTED: androgen-dependent TFPI-regulating protein-like [*Oryzias latipes*]	XP_004077548.1	4.00E-14	2.12	2.73
PREDICTED: ras-related protein Rab-11A-like [*Oreochromis niloticus*]	XP_003451264.1	4.00E-150	2.11	2.89
PREDICTED: annexin A1-like [*Oreochromis niloticus*]	XP_003440126.1	9.00E-128	2.10	2.02
PREDICTED: SH3 domain-binding glutamic acid-rich-like protein 3-like [*Takifugu rubripes*]	XP_003971708.1	6.00E-24	2.08	2.96
PREDICTED: LOW-QUALITY PROTEIN: ornithine decarboxylase antizyme 2-like [*Oreochromis niloticus*]	XP_003453914.1	2.00E-103	2.01	1.79
Microsomal glutathione S-transferase 3 [*Anoplopoma fimbria*]	ACQ58232.1	2.00E-78	2.01	1.79
Plasma retinol-binding protein 1 [*Dicentrarchus labrax*]	CBN81434.1	2.00E-112	1.97	8.71
Tetraspanin-13 [*Salmo salar*]	ACI66359.1	2.00E-64	1.94	3.14
Proteasome maturation protein [*Osmerus mordax*]	ACO09444.1	1.00E-77	1.91	1.96
PREDICTED: NADH dehydrogenase [ubiquinone] 1 beta subcomplex subunit 2, mitochondrial-like isoform 1 [*Oreochromis niloticus*]	XP_003452107.1	5.00E-57	1.89	1.91
Aldolase A [*Thunnus albacares*]	CAX62602.1	0	1.88	1.58
PREDICTED: cytochrome c oxidase subunit 7A2, mitochondrial-like [*Takifugu rubripes*]	XP_003971444.1	1.00E-29	1.82	2.02
Glutathione S-transferase(rho) [*Siniperca chuatsi*]	ACI32418.1	1.00E-102	1.82	3.62
No Hit			1.80	2.48
Ferritin heavy chain [*Lates calcarifer*]	ADU87113.1	2.00E-121	1.79	2.99
Thioredoxin [*Ictalurus punctatus*]	NP_001187021.1	8.00E-35	1.76	1.88
PREDICTED: calreticulin-like isoform 1 [*Oreochromis niloticus*]	XP_003448535.1	0	1.75	1.97
PREDICTED: cleavage and polyadenylation specificity factor subunit 4-like [*Oreochromis niloticus*]	XP_003443173.1	2.00E-162	1.72	1.91

### Gene Ontology term enrichment among pregnancy-enriched, pregnancy-depressed, and evenly expressed transcripts

**Figure 4:**
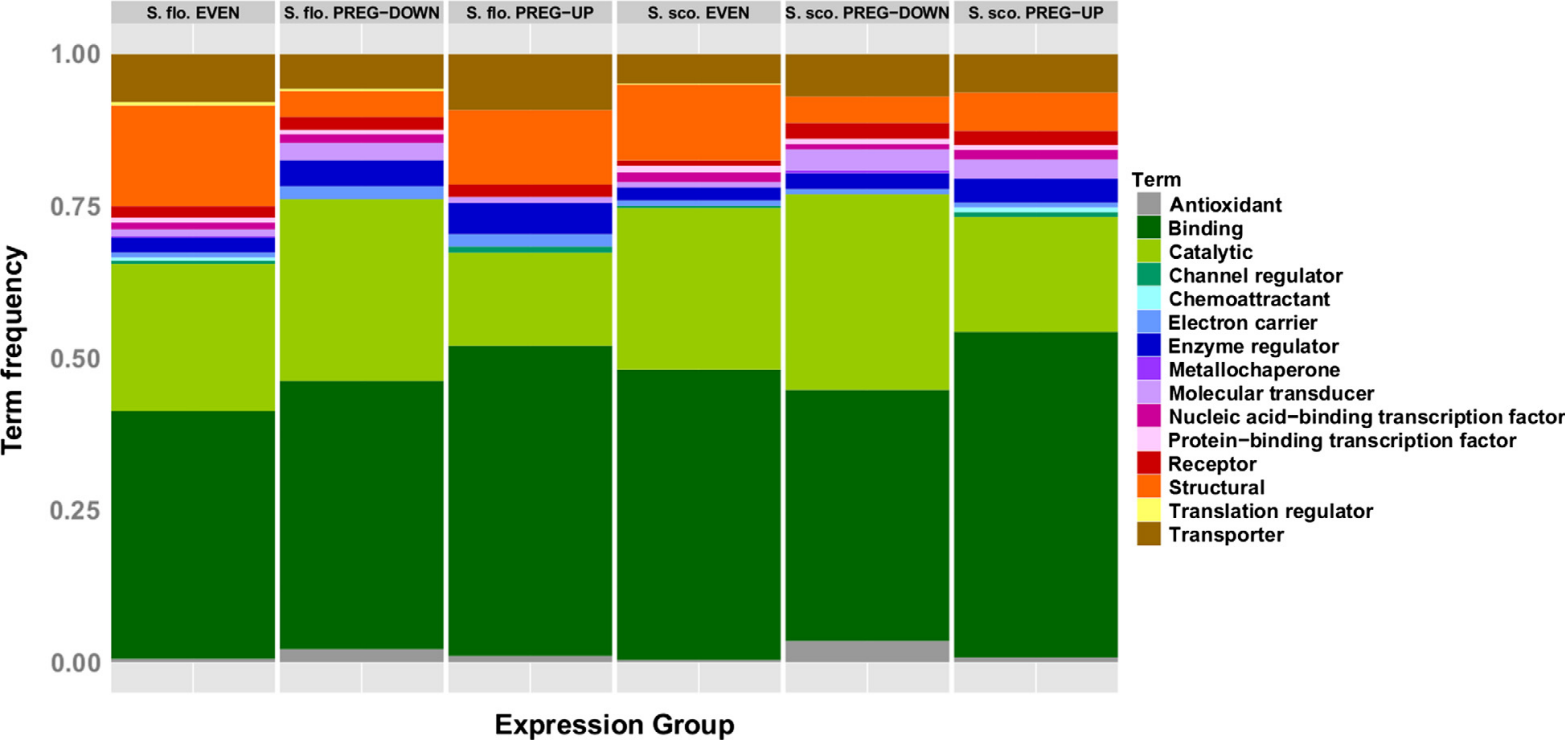
Count distributions for 15 “level 2 molecular function” GO terms across evenly expressed (EVEN), pregnancy-depressed (PREG-DOWN), and pregnancy-enriched (PREG-UP) transcript categories in *S. floridae* and *S. scovelli*. Annotations were compiled using Blast2GO.

Separate Blast2GO comparisons of the pregnancy-enriched, pregnancy-depressed, and evenly expressed contig subsets for both *S. scovelli* and *S. floridae* contigs included 15 total Gene Ontology (GO) terms (“level 2 molecular function”). These comparisons were based on the original 121 pregnancy-enriched, 207 pregnancy-depressed, and 778 evenly expressed genes in *S. scovelli*, and the 119 pregnancy-enriched, 245 pregnancy-depressed, and 296 evenly expressed genes in *S. floridae*. The relative frequencies of these terms among the different expression categories are presented graphically in Fig. 4. According to 3 × 2 tests of independence, in *S. scovelli,* “structural” (*P* = 4.180*10^−4^), “antioxidant” (*P* = 5.050*10^−3^), “catalytic” (*P* = 0.022), and “molecular transducer” (*P* = 0.034) terms are heterogeneously distributed across the three expression categories, although only the “structural” and “antioxidant” tests are robust to a false discovery rate adjustment. In *S. floridae*, “structural” (*P* = 1.720*10^−6^) and “catalytic” (*P* = 0.011) terms are heterogeneously distributed across the three expression categories, but only the “structural” difference is robust to a false discovery rate adjustment.

### Protein-coding sequence divergence of pregnancy-enriched, pregnancy-depressed, and evenly expressed genes

The ratio of nonsynonymous to synonymous substitution rates (*d*_N_/*d*_S_) differs among the three expression categories (Kruskal–Wallis test: *N*_*preg-up*_ = 19, *N*_*preg-down*_ = 66, *N*_*preg-even*_ = 46; *χ*^*2*^ = 14.138; *P* < 0.001), as does *d*_N_ alone (*N*_*preg-up*_ = 19, *N*_*preg-down*_ = 69, *N*_*preg-even*_ = 50; *χ*^*2*^ = 13.421; *P* = 0.001). The mean *d*_N_/*d*_S_ estimates of pregnancy-enriched genes (0.450) and pregnancy-depressed genes (0.413) are relatively high. In fact, they are comparable to a mean estimate of 0.505 for 79 orthologous pairs (*Drosophila simulans* and *D. melanogaster*) of male accessory-gland-enriched proteins, which we obtained from original data published by Swanson et al. (2001b). In accordance with expectations for substitutions neutral (or nearly so) in nature, we detected no significant difference in *d*_S_ among expression categories (*N*_*preg-up*_ = 19, *N*_*preg-down*_ = 69, *N*_*preg-even*_ = 50; *χ*^*2*^ = 2.780; *P* = 0.249). Figs. 7 present box-and-whisker plots of the distributions of these estimates. Pairwise, post hoc Mann–Whitney *U*-tests revealed differences in *d*_N_/*d*_S_ between pregnancy-enriched and evenly expressed (*Z* = 2.958, *P* = 0.003), between pregnancy-depressed and evenly expressed (*Z* = 3.338, *P* < 0.001), but not between the two differentially expressed categories (*Z* = 0.665, *P* = 0.506). Likewise, *d*_N_ differs between pregnancy-enriched and evenly expressed (*Z* = 2.909, *P* = 0.004), between pregnancy-depressed and evenly expressed (*Z* = 3.167, *P* = 0.002), but not between the two differentially expressed categories (*Z* = 0.9494, *P* = 0.342). We also detected a difference among expression categories in the proportion of genes with a *d*_N_/*d*_S_ estimate >0.5 (3 × 2 *G*-test of independence: *G*_*adj*_ = 7.450; *P* = 0.024).

**Figure 5:**
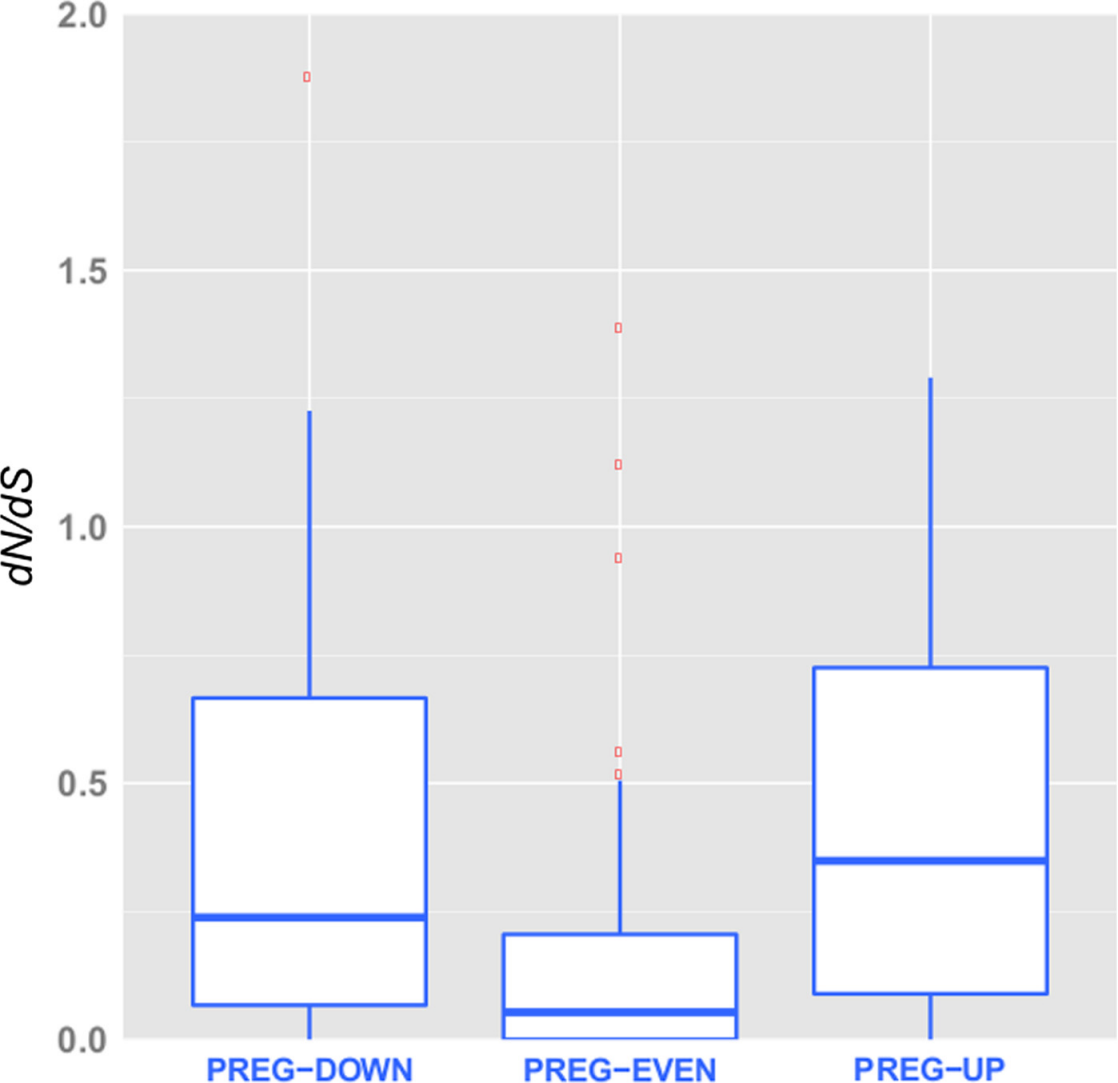
The distributions of *d*_N_/*d*_S_ for *S. scovelli*-*S. floridae* coding sequence alignments, by expression category. Standard box-and-whisker plots represent medians, upper quartiles, lower quartiles, outlier fence boundaries (whisker ends), and outliers (red rectangles). One evenly expressed observation (*d*_N_/*d*_S_ = 2.120) and one pregnancy-depressed observation (*d*_N_/*d*_S_ = 3.320) are beyond the upper boundary of the *y*-axis and consequently not visible.

**Figure 6:**
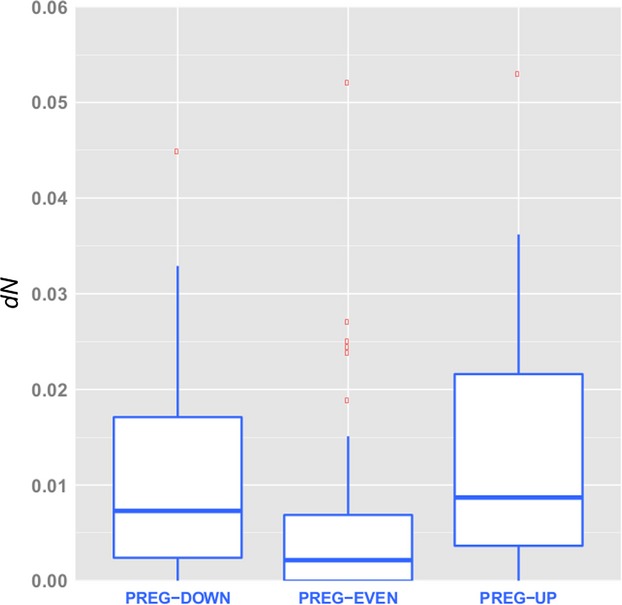
The distributions of *d*_N_ for *S. scovelli*-*S. floridae* coding sequence alignments, by expression category. Standard box-and-whisker plots represent medians, upper quartiles, lower quartiles, outlier fence boundaries (whisker ends), and outliers (red rectangles). One evenly expressed observation (*d*_N_ = 0.087) and one pregnancy-enriched observation (*d*_N_ = 0.097) are beyond the upper boundary of the *y*-axis and consequently not visible.

**Figure 7:**
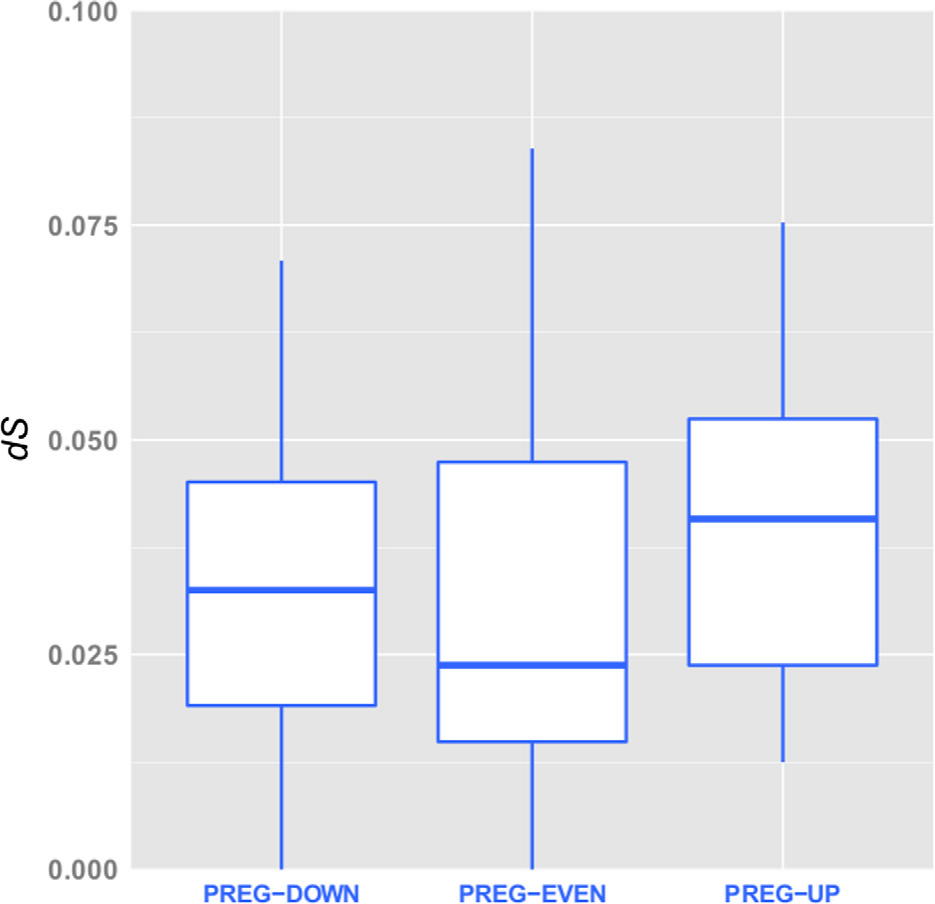
The distributions of *d*_S_ for *S. scovelli*-*S. floridae* coding sequence alignments, by expression category. Standard box-and-whisker plots represent medians, upper quartiles, lower quartiles, and outlier fence boundaries (whisker ends). One evenly expressed observation (*d*_S_ = 1.115), one pregnancy-depressed observation (*d*_S_ = 0.304), and one pregnancy-enriched observation (*d*_S_ = 0.113) are beyond the upper boundary of the *y*-axis and consequently not visible.

## Discussion

Our study marks the first application of next-generation sequencing to characterize the male pregnancy transcriptome in syngnathid fishes. Previous work used traditional Sanger sequencing to identify brood-pouch-expressed genes at large (Melamed et al. 2005) and in the narrower context of a single group of astacin-like metalloproteases (Harlin-Cognato et al. 2006). The authors of a recent study (Haase et al. 2013) used 454 and Illumina sequencing to screen the broad-nosed pipefish (*Syngnathus typhle*) transcriptome for MHC class II genes, but without regard to male pregnancy. Our initial efforts yielded just under one million 454 reads between *S. scovelli* and *S. floridae*, but we were nevertheless able to assemble these data into hundreds of putative transcripts at least 1000 nt in length (1816 such transcripts for *S. scovelli* and 812 for *S. floridae*). Contig length did not differ among different expression categories, so the assemblies themselves likely did not hamper our ability to identify pregnancy-enriched, pregnancy-depressed, and evenly expressed transcripts. The superiority of the *S. scovelli* transcriptome assembly is most likely due to greater sequencing depth (approximately 1.7 times that of *S. floridae*), as recent work has demonstrated that the relationship between sequencing effort and gene discovery for single-tissue transcriptome assemblies begins to asymptote at approximately 20 million short reads (Francis et al. 2013). Our assemblies, therefore, have most likely missed a number of transcripts expressed in the male brooding tissues of these species, especially those which exist at low abundance. Nevertheless, our assemblies allowed us to identify over 5000 putative transcript orthologs (over 1000 represented by at least 10 reads), which we consider ample fodder for a descriptive study of the molecular evolution of putative male pregnancy genes in the *Syngnathus* lineage.

Two clear limitations of our current study, particularly regarding differential expression, are the lack of biological replication and the relatively shallow depth of sequencing coverage. Pooling of individual RNA samples was necessary to obtain sufficient materials for the type of cDNA library preparation employed in our study, but the latest RNA-seq technologies render this a constraint of the past. We attempted to make our definitions of differential expression categories more stringent based on intersections between results from *S. scovelli* and *S. floridae*, but we realize that our inferences are subject to change in light of future results based on biological replicates. The depth of sequencing coverage in the current study is also a reflection of the technological resources available at the time the data were generated. Indeed, a minimum of 10 million short reads per individual is commonly considered necessary to reliably detect signals of differential expression among sampling noise (Wang et al. 2011). Therefore, while our “differentially expressed” genes represented by hundreds or thousands of mapped reads are likely to be reliable, those contigs in our data set with fewer mapped reads should be interpreted more cautiously (See Table S3).

### Functional similarity among pregnancy-enriched, pregnancy-depressed, and evenly expressed transcripts

Functional annotation by way of Gene Ontology terms revealed subtle differences among expression categories for both pipefish species. We generally found GO term distributions to be quite similar across expression categories and between species (Fig. 4), with a few notable exceptions. “Structural” GO terms appear to be higher in frequency in the evenly expressed group for *S. scovelli*, relative to the differentially expressed groups. This observation is perhaps not surprising, as structural constituents of ribosomes, epidermis, the cytoskeleton, and other intra- and extracellular components should be necessary for routine biological function of brooding tissues regardless of pregnancy. For both species, transcripts annotated as “catalytic” in activity appear to be underrepresented in the pregnancy-enriched category of genes, relative to pregnancy-depressed and evenly expressed groups. Why enzymes in general should be underrepresented among transcripts that are especially abundant during pregnancy is unclear. Equally inexplicable is why *S. scovelli* transcripts characterized by antioxidant activity are overrepresented in the pregnancy-depressed group, and those with molecular transducer activity are overrepresented in the differentially expressed groups. Without a better understanding of the cellular and biochemical changes associated with male pregnancy, it is difficult to speculate on whether these differences are in line with biological expectations.

### Toward candidate “male pregnancy” genes

We used between-species agreement, at the levels of statistical significance and expression bias direction, to identify a preliminary list of genes up- and down-regulated with respect to pregnancy in the male brooding tissues of *Syngnathus* pipefishes. As mentioned, our reliance on pooled RNA-seq libraries and cross referencing between *S. scovelli* and *S. floridae* in the current study is not an ideal experimental design. However, the highly covered contigs with very disparate numbers of mapped reads from pregnant and nonpregnant libraries most likely represent genes regulated as a function of male pregnancy status (Tables 1 and 2). Our confidence in these genes playing conserved, pregnancy-associated roles within this particular pipefish lineage is strengthened by the high between-species correlation of pregnant-to-nonpregnant expression ratios (Fig. 3), but again, these results should be considered suggestive. Also worth noting is the relationship between total read coverage and expression category. Specifically, more total sequencing reads were mapped to pregnancy-enriched transcripts than evenly expressed transcripts. This is perhaps expected given a handful of genes that must presumably be up-regulated by several orders of magnitude to facilitate an involved process such as pregnancy, but the pattern also suggests that highly expressed genes from other nonreproductive tissues should be used in future comparisons with male pregnancy genes to control for gene-specific transcription level in general.

Several annotated transcripts from our assemblies appear to be highly expressed in pregnant relative to nonpregnant male brooding tissues. For example, a transcript with high sequence similarity to a protein regulated by estrogen (ACX94453.1) in gilthead seabream is approximately 100-fold more abundant in pregnant male libraries. A previous study reported that exposure to a synthetic estrogen (17α-ethinylestradiol) reduces the ability of *S. scovelli* males to become pregnant (Partridge et al. 2010), so there are now multiple reasons to believe that estrogen-sensitive molecules affect the biology of male pregnancy. An apparent chitinase-3 homolog, an astacin-like metalloprotease, an antimicrobial peptide, and a major component of a fish-derived hemotoxin are also all pregnancy-enriched, at fold changes of five or greater. Vertebrate chitinases have been associated with innate and adaptive immune responses as well as tissue remodeling in mammals (Lee et al. 2011), but there is no known basis for a specific role in male pregnancy. The metalloprotease gene patristacin was previously sequenced and characterized as pregnancy-enriched in the male *S. scovelli* brood pouch (Harlin-Cognato et al. 2006), so our results support the notion that this gene plays an important role in male pregnancy. The up-regulation of antimicrobial genes during pregnancy could reflect an innate immune response to safeguard recently transferred eggs or developing embryos from bacterial infection, but this idea awaits experimental verification. Why a homolog of venom components from scorpaeniform fishes (Ghadessy et al. 1996; Ueda et al. 2006) is pregnancy-enriched, and whether the pipefish ortholog retains similar biochemical properties, are both unclear.

We detected more than twice as many pregnancy-depressed as pregnancy-enriched genes in *S. scovelli* and *S. floridae*. One possible explanation for this disparity is that large-scale transcriptional suppression of the paternal immune system during pregnancy occurs to prevent embryonic rejection, a phenomenon reported to occur in placental mammals (Moffett and Loke 2006). Our GO analysis at the “biological processes” term level, however, provides no evidence of an over-representation of immune function terms among pregnancy-depressed genes for either species. Notably, several pregnancy-depressed genes demonstrate extremely large fold changes. Interestingly, an apparent paralog of patristacin is more than 800-fold down-regulated in pregnant male brooding tissues. Recall that patristacin is a pregnancy-enriched astacin-like metalloprotease, presumed to have been coopted (from liver and/or kidney) for expression in the brood pouch (Harlin-Cognato et al. 2006). Our *S. scovelli* pregnancy-depressed patristacin-like protein is actually quite divergent (68.7% amino acid identity) from the *S. scovelli* brood-pouch patristacin published by Harlin-Cognato et al., while our *S. scovelli* pregnancy-enriched patristacin is identical in amino acid sequence to the published molecule. Therefore, we suspect that the role of patristacins in male pregnancy is more complicated than previously understood and appears to involve drastic temporal differences in expression among paralogs.

Gene-family-wide roles in male pregnancy are not likely restricted to astacins, as multiple C-type lectin paralogs are pregnancy-enriched (two) and pregnancy-depressed (three) according to our results. C-type lectins are calcium-dependent, sugar-binding proteins, thought to perform a wide range of biochemical functions, including cell adhesion, endocytosis, and host-pathogen interaction (Dodd and Drickamer 2001). Authors of a previous study produced a cDNA library generated from male brooding tissues of the tiger tail seahorse (*Hippocampus comes*) using conventional sequencing methods (Melamed et al. 2005). Approximately 15% of the 250 clones sequenced by Melamed et al. correspond to c-type lectin-like sequences, which the authors assembled into three distinct unigenes. Melamend et al. confirmed the presence of one c-type lectin (“*hc* CTL III”) in the fluid of the pouch during pregnancy via Western blot. Furthermore, the authors cloned this molecule into an *E. coli* expression vector and confirmed both antimicrobial activity and mouse red blood cell agglutination in vitro. Given the transcriptional profiles from our study and the above functional information, it seems likely that c-type lectins are biochemically relevant to processes that take place within the syngnathid brood pouch. Future sequence-based genomic studies to characterize the evolutionary relationships among lectins, along with refined functional assays, will be required to elucidate the precise roles these molecules have played during the evolution of male pregnancy.

### Protein-coding divergence of male pregnancy genes

Our results suggest that male brooding tissue proteins expressed at high and low levels in pregnant relative to nonpregnant individuals have diverged more rapidly than those not differentially expressed (Fig. 6), an observation consistent with the phenomenon of rapidly evolving proteins expressed in the reproductive tissues of other animal taxa (Wyckoff et al. 2000; Good and Nachman 2005; Artieri et al. 2008; Walters and Harrison 2010; reviewed in Swanson and Vacquier 2002a,b). The ratio of nonsynonymous to synonymous rates of substitution is also elevated for pregnancy-enriched and pregnancy-depressed genes, which indicates differences in the nature of selection on these groups of genes since divergence of *S. scovelli* from *S. floridae* (Fig. 5). As expected, we found no evidence for a difference in the synonymous substitution rate (*d*_S_) among expression categories, so differences in selection on codon usage (Shields et al. 1988) as a function of pregnancy expression status are not apparent.

Elevated *d*_N_ and *d*_N_/*d*_S_ for differentially expressed genes may reflect historically important functional roles of male pregnancy linked to fitness in one or both lineages and therefore divergence by positive selection. For example, it is now well documented that males regulate the osmotic (Ripley 2009) and nutritional (Ripley and Foran 2009; Kvarnemo et al. 2011) environments of gestating embryos. Our data also suggest that some genes differentially regulated in the male brooding tissues during pregnancy are homologous to molecules that mitigate pathogenesis in other vertebrates, specific examples being c-type lectins (Melamed et al. 2005) and a bactericidal permeability-increasing protein (Nam et al. 2010). In addition, embryo survivorship in the brood pouch appears to be a function of maternal phenotype (Paczolt and Jones 2010; Mobley et al. 2011). If male brooding tissues mediate this relationship at any level, lineage differences in the strength of sexual selection may affect evolutionary trajectories of brood-pouch biology across syngnathid taxa. This type of consequence is expected in the case of *S. scovelli* and *S. floridae*, given that they demonstrate markedly different mating systems (Jones and Avise 1997a,b). We also found that a higher proportion of differentially expressed relative to evenly expressed genes is characterized by a *d*_N_/*d*_S_ ratio >0.5. Although a *d*_N_/*d*_S_ value ≤1.0 is not consistent with positive selection *sensu stricto*, gene-wide estimates >0.5 are often considered tentatively as signatures of positive selection at a small number of residues amidst purifying selection across the remainder of the protein (Swanson et al. 2001b, 2004).

Another equally plausible scenario, however, is that pregnancy-enriched and pregnancy-depressed genes have simply been less constrained by purifying selection than have genes from the evenly expressed group. Genes expressed evenly across multiple tissues, for example, often perform housekeeping functions and commonly demonstrate depressed nonsynonymous substitution rates relative to those characterized by more promiscuous expression patterns (Duret and Mouchiroud 2000; Zhang and Li 2004). Distinguishing between these two alternatives ultimately requires better taxonomic sampling for formal, more powerful tests of positive selection (Nielsen and Yang 1998; Zhang et al. 2005) and coding sequence information from the transcriptomes of other tissues both reproductive and nonreproductive, measures we are currently pursuing with additional data.

### Transcriptomic insights into comparative and functional genomics of novel, complex traits

An increasing number of studies have taken advantage of next-generation sequencing technologies to profile transcriptomes associated with ecologically interesting tissues and traits in organisms with few genetic resources. Recent examples include regenerative tissue in newts (Looso et al. 2013), the venom gland in rattlesnakes (Rokyta et al. 2011), Antarctic icefish skeletal muscle (Coppe et al. 2013), and uterine tissue from pregnant and nonpregnant, live-bearing skinks (Brandley et al. 2012). It is clear that RNA-seq approaches constitute a very powerful tool for understanding the molecular underpinnings of novel adaptations, especially when differential expression information can be combined with sequencing data across multiple lineages. Here, we have performed a preliminary analysis to identify transcripts associated with the phenomenon of male pregnancy in syngnathid fishes and measure sequence coding divergence of these genes between two species. Specific gene families, including astacin metalloproteases and c-type lectins, appear to vary transcriptionally as a consequence of male pregnancy, but a better understanding of their precise evolutionary histories and ongoing roles in the brood pouch is contingent on additional comparative information. We also found that, like other classes of reproductive genes, pregnancy-enriched and pregnancy-depressed proteins are especially likely to have experienced ongoing positive selection. The adaptive nature of the mechanisms responsible for this pattern remains uncertain, but future applications of our current approach on a larger scale, with improved sequencing coverage, promise important evolutionary insights into a truly original example of phenotypic novelty.

## Data Accessibility

All raw 454 sequencing read files have been deposited in the NCBI Sequencing Read Archive (SRA) and will be available through BioProject IDs PRJNA197211 (*S. scovelli*) and PRJNA198438 (*S. floridae*).

## Supporting Information

**Figures S1** and **S2** corresponding to *S. scovelli* and *S. floridae*, respectively, are MA-plots from DEG-seq differential expression analyses. M (*y*-axis values) = log_2_*C*_*Pregnant*_ – log_2_*C*_*Nonpregnant*_, where *C*_*i*_ represents the number of uniquely mapped reads from library *i*. A (*x*-axis values) = (log_2_*C*_*Pregnant*_ + log_2_*C*_*Nonpregnant*_)/2. Genes (contigs) for which the DEG-seq *P*-value < 0.05 are represented by red dots on the MA-plots.

**Table S1** contains 6 columns, which correspond to *S. scovelli* contig name, contig length, number of uniquely mapped reads from the pregnant library, number of uniquely mapped reads from the nonpregnant library, the normalized pregnant/nonpregnant fold change, and the DEG-seq “MARS” *P*-value for differential expression.

**Table S2** contains the same information, but for *S. floridae*.

**Table S3** contains this information, including top blastx hit descriptions, for the 150 orthologous pairs that belong to pregnancy-enriched, pregnancy-depressed, and evenly expressed categories.
