# Supplementary figures and images for: Functional similarity and molecular divergence of a novel reproductive transcriptome in two male-pregnant Syngnathus pipefish species
Source: Ecol Evol. 2013 Sep 20;3(12):4092–108. doi: 10.1002/ece3.763 (PMC3853555; doi:10.1002/ece3.763)

Pregnant VS Nonpregnant

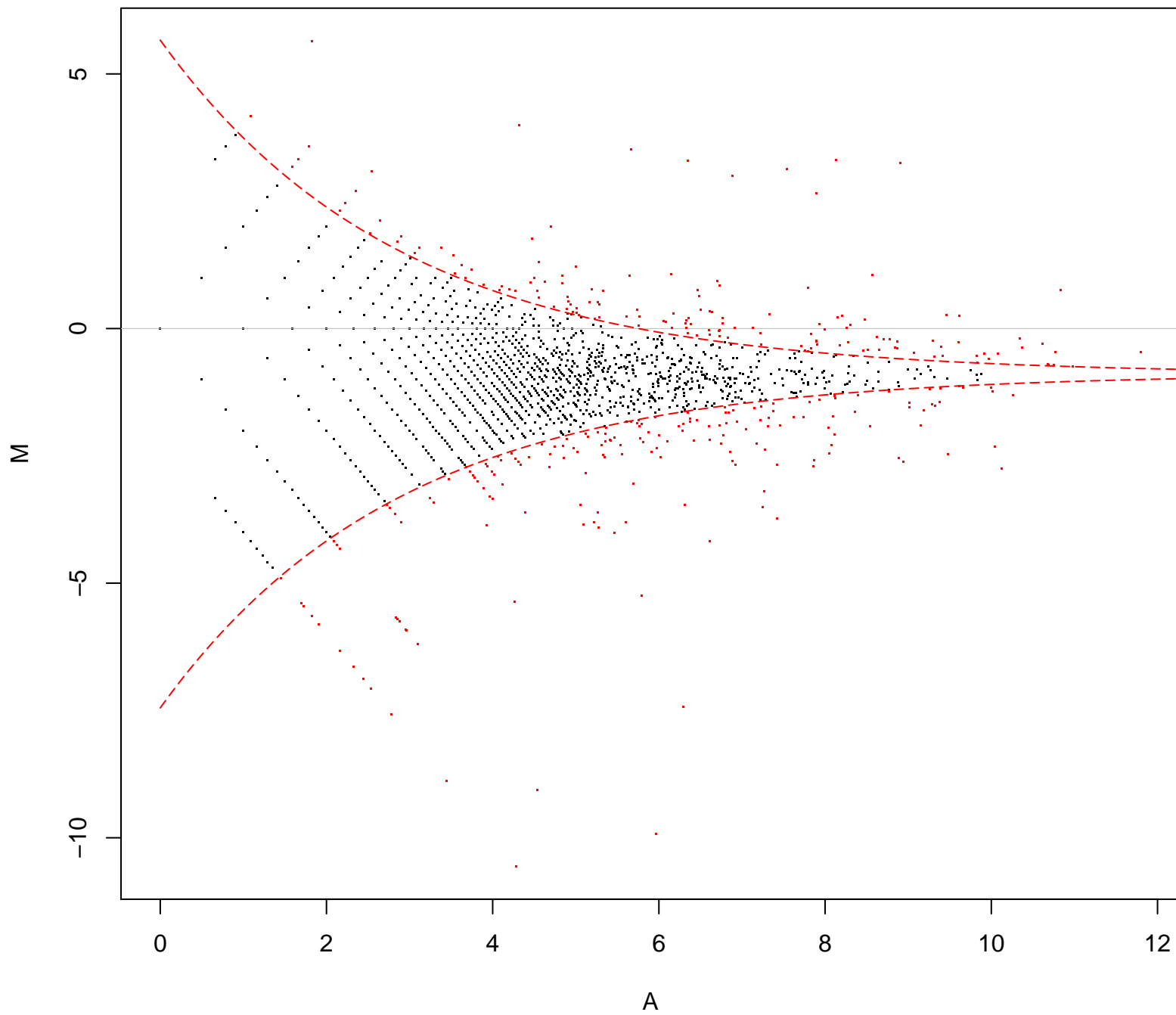

Supplement: Supplementary file 1 [file ece30003-4092-SD1.pdf]

Pregnant VS Nonpregnant

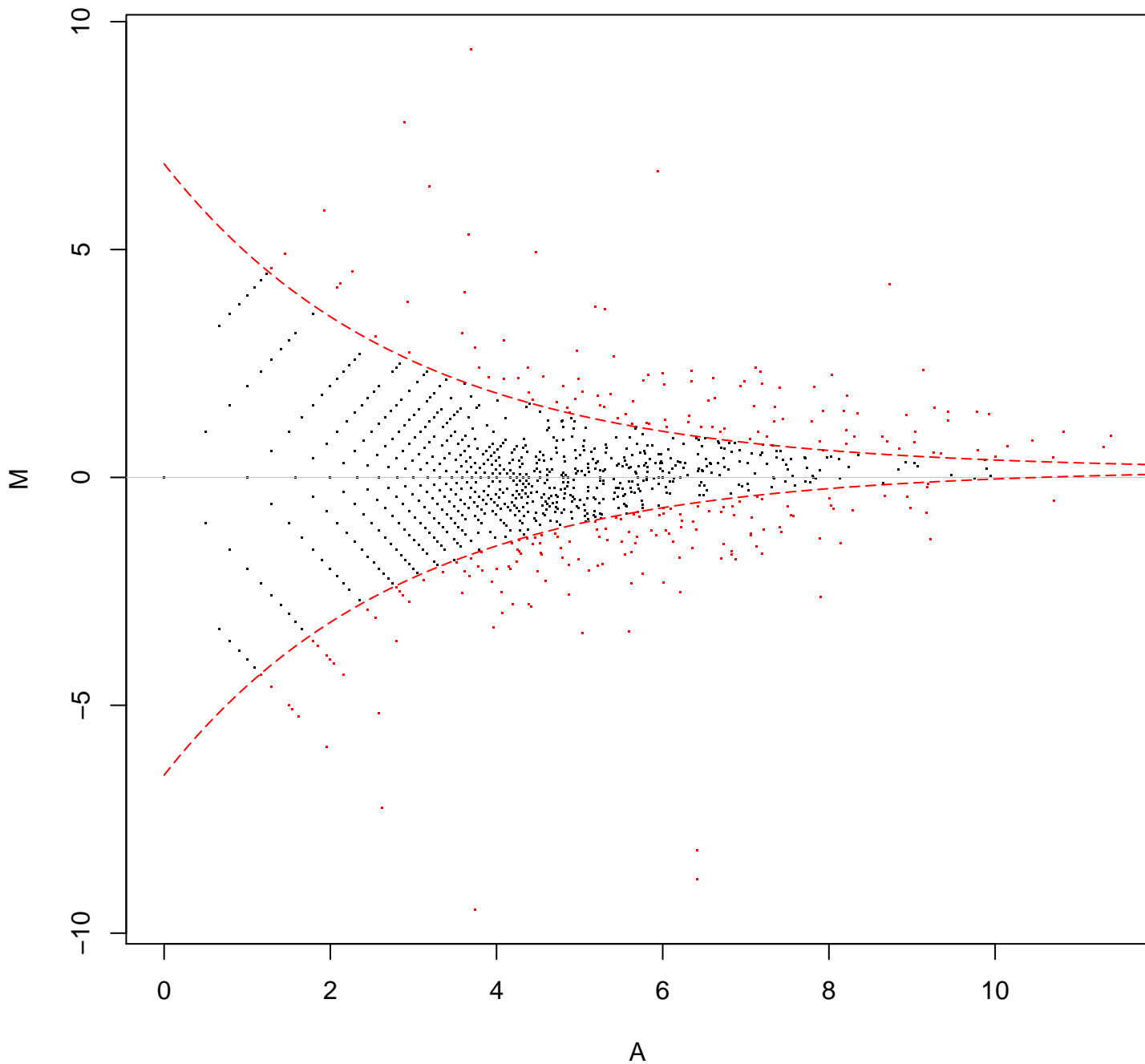

Supplement: Supplementary file 2 [file ece30003-4092-SD2.pdf]
